# Stiffness Identification of Foamed Asphalt Mixtures with Cement, Evaluated in Laboratory and In Situ in Road Pavements

**DOI:** 10.3390/ma13051128

**Published:** 2020-03-03

**Authors:** Lukasz Skotnicki, Jarosław Kuźniewski, Antoni Szydlo

**Affiliations:** Roads and Airports Department, Faculty of Civil Engineering, Wroclaw University of Science and Technology, 50-370 Wroclaw, Poland; jaroslaw.kuzniewski@pwr.edu.pl (J.K.); antoni.szydlo@pwr.edu.pl (A.S.)

**Keywords:** recycling, foamed asphalt mixtures with cement (FAC), base layer, reclaimed asphalt pavement (RAP), fatigue durability

## Abstract

The article presents the possibilities of using foamed asphalt in the recycling process to produce the base layer of road pavement constructions in Polish conditions. Foamed asphalt was combined with reclaimed asphalt pavement (RAP) and hydraulic binder (cement). Foamed asphalt mixtures with cement (FAC) were made, based on these ingredients. To reduce stiffness and cracking in the base layer, foamed asphalt (FA) was additionally used in the analyzed mixes containing cement. The laboratory analyzes allowed to estimate the stiffness and fatigue durability of the conglomerate. In the experimental section, measurements of deflections are made, modules of pavement layers are calculated, and their fatigue durability is determined. As a result of the research, new fatigue criteria for FAC mixtures and correlation factors of stiffness modules and fatigue durability in situ with the results of laboratory tests are developed. It is anticipated that FAC recycling technology will provide durable and safe road pavements.

## 1. Introduction

Recycling of road pavements makes it possible to reuse road materials that have been refined with binders such as asphalt or cement. The main reason for recycling is the decrease in the availability of stone raw materials, the reduction of aggregate transport costs, and thus the relief of the road and rail network, as well as the liquidation of landfills from damaged road pavements. One of the recycling solutions is the use of foamed asphalt as a binder for recycled aggregates. Foamed asphalt is created by injection through a binder nozzle heated to a temperature of approximately 170 °C with the addition of water, as a result of which, the volume of asphalt increases 15 to 25 times, which in turn allows the smallest grains of the mix to be surrounded. The optimum amount of water for foaming, depending on the type of binder, ranges from 2.0%–3.5% [1,2,3]. There are also attempts to use ethanol instead of water to foam the asphalt binder [4].

Foamed asphalt (FA) is used in cold, deep recycling technology during the modernization of road pavement construction and in modern technology of warm mix asphalt (WMA), in which the production temperature of asphalt materials is reduced by about 50 °C compared to traditional production technology of hot mix asphalt (HMA). 

All over the world, attempts are made to use foamed asphalt in road engineering. Foamed asphalt is used primarily through recycling to make the sub-base layers of road pavement constructions. According to [5], tests have shown that the stiffness modulus of the mixture with foamed asphalt depends on both the stress state and the test temperature. On the basis of triaxial tests, it was found that for mixes without active filler, the hardening of the mixture is generally independent of temperature. According to the Marshall stability results, the water content of the foam has no significant influence on the performance of the foam asphalt mixture [6]. The results from the study [7] suggest that foamed asphalt cold recycling mixtures have a high modulus and small temperature shrinkage stress, reducing early damage caused by pavement cracks.

In Saudi Arabia, foamed asphalt is mainly used for the production of the sub-base layer and upper base layer, made of reclaimed asphalt pavement (RAP) [1]. In foamed asphalt production processes, the asphalt of high penetration 160/220 is often used [8].

In Indonesia, attempts are being made to use foamed asphalt in asphalt concretes containing only a mineral mixture. Foamed asphalt replaces the regular road binder [9]. These asphalt mixtures based on foamed asphalt can be used in the upper base layer of road pavements.

Mixtures with RAP can be beneficial to the moisture resistance of warm mix asphalt (WMA) and hot mix asphalt (HMA) mixtures. Moisture resistance of asphalt mixtures increases with the increase in RAP content [10]. In addition, the results presented in [11] indicate that the foam processing slightly reduced high-temperature performance and temperature sensitivity while improving the resistance for fatigue cracking.

In the USA, foamed asphalt is commonly used for construction layers of road pavements. An innovative solution is the use of foamed asphalt to stabilize foundations based on ashes [12]. In the USA, in Johnson County, Iowa, RAP temperatures were found to have a significant effect on indirect wet tensile strength, asphalt foam blends produced in the cold recycling site. As the RAP temperature increased, the optimal foam asphalt content decreased—this is due to the activation of the asphalt from waste at a higher temperature and facilitating compaction [13]. Furthermore, the type of asphalt binder contained in the recovered asphalt material has a significant impact on the change in the complex modulus of the recycled mixture [14].

During the 8th Conference on Asphalt Pavements for Southern Africa in Sun City, the authors of [15] and the authors of the research presented in [1] showed an increase in indirect tensile strength (ITS) along with an increase in cement content. Additionally, mixtures containing foamed asphalt showed higher strength values (ITS) than mixtures containing asphalt emulsion. For the same mixtures, a similar relationship was observed in determining the stiffness modulus [16]. Furthermore, the authors’ experience show that mixtures containing foamed asphalt have higher durability than mixtures containing asphalt emulsion. This is caused by the different properties of these binders.

The content of asphalt binder has a significant effect on the wet and dry ITS values of materials stabilized with foamed asphalt [17] but a smaller effect on materials stabilized with asphalt emulsion [1,2,15].

Due to the climatic conditions in Central European countries, road pavements should be water- and frost-resistant. On the basis of the research of recycled foamed asphalt pavement, it was found that the use of foamed asphalt improves its tensile stress strength and the mechanical properties of the pavement [18,19,20,21]. Moreover, the use of foamed asphalt in mixtures ensures higher water and frost resistance, higher creep stiffness modulus, and higher resistance to plastic strain than when using asphalt emulsion [2,19]. 

The optimal content of foamed asphalt and hydraulic binder (Portland cement) for mixtures of the base layer gives the desired physical (the air void content) and mechanical parameters (wet-dry ITS) [22,23,24].

In the test section on the heavily trafficked Greek highway pavement presented in [24], the results of strain and deformation in a layer made of foamed asphalt and recycled material showed that the critical in situ stress in the FA layer was lower than the maximum expected tensile stress threshold. This fact indicates the improvement of fatigue properties of this type of mixture. 

Water-based disintegration asphalt emulsions are mainly used for the recycling of asphalt layers in Poland. As a result of mixing reclaimed asphalt pavement (RAP), cement binder and asphalt emulsion, a so-called mineral cement emulsion mixture (MCE) is created. Innovative use of foamed asphalt in mineral-cement mixtures (FAC) in exchange for asphalt emulsion may have a positive effect on the properties of renovated road pavements and the process of building them. 

The article presents alternative possibilities for using foamed asphalt to produce the base layer. Foamed asphalt was combined with a mineral mix (reclaimed asphalt pavement (RAP) + possible material for improving gradation) and a hydraulic binder (cement). On the basis of these ingredients, foamed asphalt mixtures with cement were made and marked with the symbol FAC.

## 2. Materials and Methods

Materials from recycled degraded pavement (test section) were used in the research process. For this purpose, RAP was used from degraded wearing and base course layers as well as crushed granite stone from the base.

Based on control laboratory tests of density, bulk density, Marshall stability, and flow, the technology of production (composition design) of the FAC mixtures was proposed. The durability of the future road pavement is significantly affected by the stiffness and fatigue life of the FAC mixture. Stiffness and fatigue durability in laboratory conditions were determined for FAC-type mixtures. Then, reconstruction of the recycled pavement layers and implementation of the experimental sectionߣs pavement layers began. Foamed asphalt, with the addition of cement binder to the base layer, was used. Nevertheless, the presence of too rigid mixtures in the base layers can cause the formation of shrinkage cracks, which copy to the pavement layers of asphalt mixtures in the form of reflected cracks. The use of foamed asphalt in combination with cement allowed the limitation of stiffness and shrinkage cracking of the mixture in terms of its use in the base of the road pavement.

**Figure 1:**
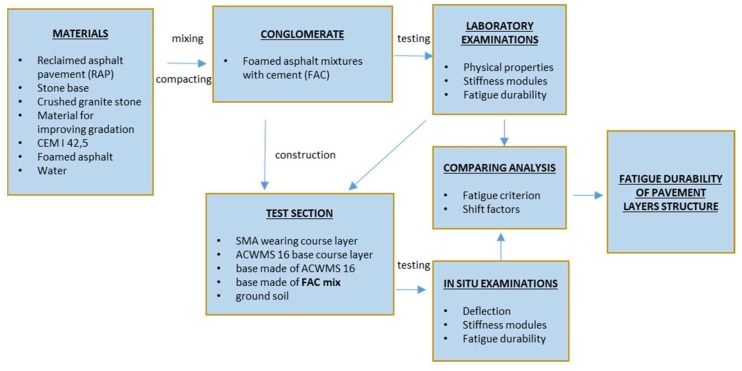
Research methodology flow chart.

Asphalt layers (asphalt concrete and SMA) were laid on the base layer of the test section made of the FAC mixture. After making the pavement on the experimental section, deflection measurements were made, pavement layer modules were determined, and its fatigue durability was determined. As a result of research, an attempt was made to correlate the stiffness and fatigue life determined in the laboratory with the parameters of FAC mixtures of the test section. New fatigue criteria have been introduced for FAC mixtures used in the base layers. Laboratory tests of the stiffness modulus and fatigue life and developed fatigue criteria can be used to estimate the durability of future road pavement constructions, based on base layers of FAC mixes. The flow chart of the research approach is shown in Figure 1.

The design of the mixture should be correlated with the design of the pavement construction and the organization of works, depending on the method of its implementation. The procedure for designing the composition of the FAC mixture for rebuilding an existing road requires the following steps:

- recognition of pavement structure and layer properties on the basis of samples drilled from it, together with material taken from the base,
  − type and group determination of bearing capacity of the base,
  − determination of the thickness and type of structural layers of the old pavement,
  − recognition of the material forming particular layers,
  − marking of the old binder content in bituminous layers,
- formulation of recycling conditions—method of preparing the mixture, determination of base thickness,
- preparation of analytical samples, performance of tests related to the development of a recipe and determination of the physical and strength characteristics of the designed FAC mixture.

The mineral mixture may consist of the recycled asphalt (RA) material itself obtained directly from milling the pavement or from crushing the lumps from the demolition of the pavement, if it meets the requirements for grading according to [25]. Without this condition, the RA material should be improved with a mineral aggregate.

In the research process, materials from the recycling of the degraded pavement (test section) were used in the form of RAP + stone material from the base + 0/31.5 mm material for improving the mixture gradation of the igneous rock fraction—gabbro. Grading boundary curves for mineral cement emulsion mixtures (MCE) were adapted for research purposes [25]. The recovered mixture (with a content of 5.05% asphalt) did not meet the conditions for gradation, therefore the material for improving gradation was used—0/31.5 mm of igneous rock—gabbro. Table 1 describes the grading of the mineral mixture, taking into consideration the grain size and the percentages of individual components.

The optimal cement addition was estimated on the basis of the compressive strength test at various foamed asphalt contents, according to [26]. FAC mixtures embedded in the base layers should be characterized by susceptibility to deformation on the one hand, and rigidity associated with the transmission of strains from higher layers on the other. The use of cement in typical asphalt mixtures is usually limited to 6% [27], which is why laboratory tests during this work for FAC mixtures were carried out with cement content of 2.38%, 3.38%, and 4.38%.

The optimal water content needed to foam asphalt binder is about 2–3% [1,2,3,28]. According to [29], in a foaming process, the injection of higher foaming water content (FWC) results in a higher volume expansion but lower stability of foamed asphalt at a certain foaming temperature and air pressure. The amount of water used for foaming the asphalt allows optimal foaming of the binder in the amount of 2.5%.

Due to the cement binder present in the mixture, additional water content was necessary for its proper compacting and setting. The water content of the mineral mix with cement, guaranteeing its maximum compaction, was determined on the basis of optimization according to Proctor methods—method II, based on [30]. The optimum moisture content of the mix was 6.35%.

The FAC mixtures use Nynas Nyfoam 190 of high penetration asphalt with a penetration of 160 –220 [31]. Estimating the content of asphalt binder and allowing for the maximum stability of the mixture is possible on the basis of the Marshall test [32]. However, FAC mixtures should not be too susceptible to deformation, but also not too stiff, because of the possibility of cracking. This condition was adopted, due to the need to reduce the shrinkage of the mixture and the formation of cracks in it that could copy into the upper layers of the road pavement. For this stability, the percentage content of foamed asphalt was estimated, which should be added to the mixture. Two levels of asphalt content of 3.5% and 5.5% were used in the analyzed mixtures.

Based on the optimization of foamed asphalt and cement content, a laboratory composition of FAC mixtures was proposed, see Table 2.

In order to reduce pavement damage and increase its durability, it is important to verify the material parameters of individual pavement layers and carry out the necessary tests, depending on the operating conditions of these materials. As part of the study, FAC mixtures were tested (Table 2), which were applied to the base layers of the pavement.

The qualitative evaluation of the proposed mixtures, collected from recycled old road pavements, consisted of several compatibility tests. Analyzes included in the research program are shown in Table 3.

All laboratory samples were compacted using the Marshall method with 75 blows per side [25]. The presented parameters of the FAC mixture are necessary for the correct execution and compaction of the road pavement layer of the test section. The proper load-bearing capacity of the base layer determines the increased durability of the entire road pavement construction. 

The main research element were analyzes of stiffness and fatigue life of FAC mixtures. The bending tests of the 4-point beam (4PB-PR) were used to perform them:

- the complex modulus was determined according to [36] at −10 °C, +10 °C, +30 °C, and +55 °C,
- fatigue life according to [37] at +10 °C.

**Figure 2:**
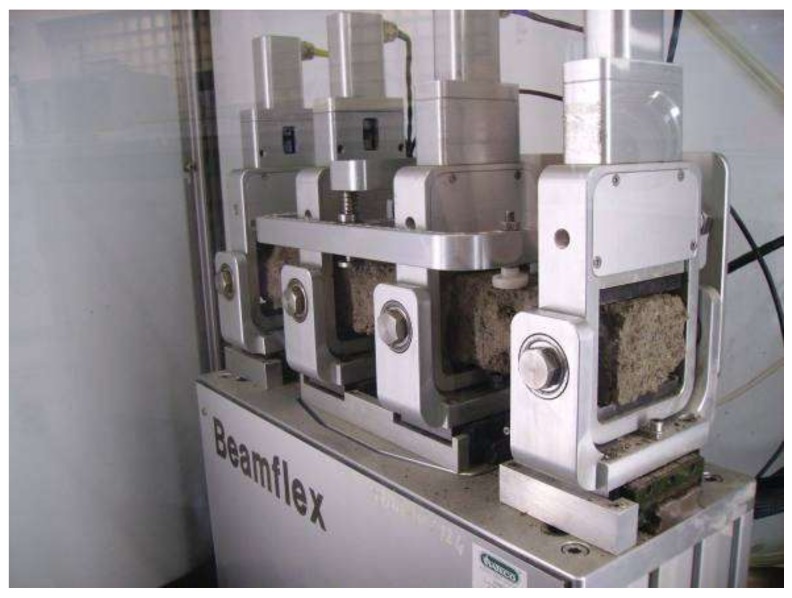
Beam-Flex apparatus.

**Figure 3:**
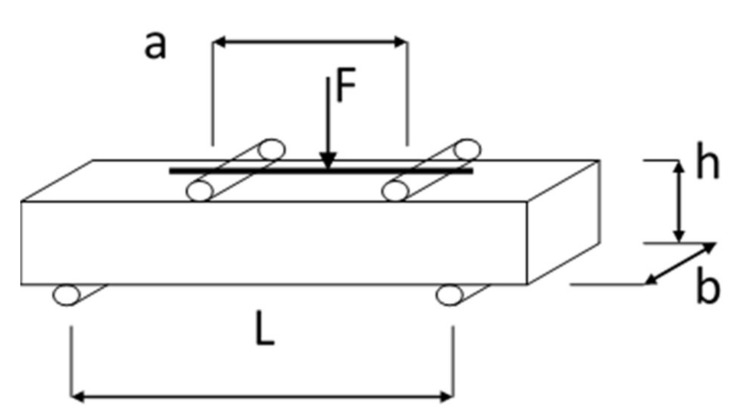
Load diagram for FAC mixture samples in the 4PB test.

The loading frequency in 4PB-PR tests was 10 Hz. The device for testing stiffness and fatigue life is shown in Figure 2. The fatigue machine allows for simultaneous testing of changes in stiffness of the material being analyzed, determining the so-called complex modulus. The tests use prismatic beams with nominal dimensions: effective length (beam span between supports) L = 357 mm, b = 60 mm, h = 50 mm, as seen in Figure 3.

The study of the complex modulus of FAC mixture stiffness was carried out by the method of permanent deformation ε = 50 × 10^−6^ m/m. During fatigue tests, using the permanent deformation method, 5 load levels were adopted in the form of given strains: ε = 500 × 10^−6^ m/m, ε = 400 × 10^−6^ m/m, ε = 200 × 10^−6^ m/m, ε = 100 × 10^−6^ m/m, ε = 50 × 10^−6^ m/m. The number of load cycles N_f/50_ was recorded until the complex stiffness modulus dropped to 50% of the initial value—conventional fatigue criterion.

After the laboratory research, the trial field phase was implemented. The FAC mixtures designed in the laboratory were built into the base layer of the road pavement section. The stiffness modulus and fatigue durability of FAC mixtures layer were estimated. Results of laboratory examinations were compared with results of bearing capacity of this test section.

## 3. Results and Discussion

### 3.1. Basic Research

The designed FAC mixtures are intended for the lower base layer of the test section pavement. Tests were conducted to determine the basic properties of the mixtures on density, bulk density, air void content, Marshall stability, flow and compressive strength for the FAC mixture used. Base on the optimisation process the mixture C3A3 was choosen for further analysis and for applying to trial field phase. Results from the laboratory tests and analyses carried out for optimal mixture C3A3 are given in Table 4. The results given in Table 4 are mean values calculated from a minimum of three representative samples for each feature. 

The compressive strength of samples from the FAC mixture increases as the curing period increases and takes values from 1.5 to 2.5 MPa over 7 to 28 days. This is influenced by the presence of the cement added and its hydration time in the mixture. 

The Marshall stability in FAC mixtures has a similar relationship to compressive strength. Its value increases with the length of the sampleߣs “life” in the range of 10.0–12.5 kN. The increase in stability value and decrease in deformation value over time suggests a significant effect of cement presence added to the mixture.

### 3.2. Stiffness and Fatigue Durability in Laboratory Conditions

**Figure 4:**
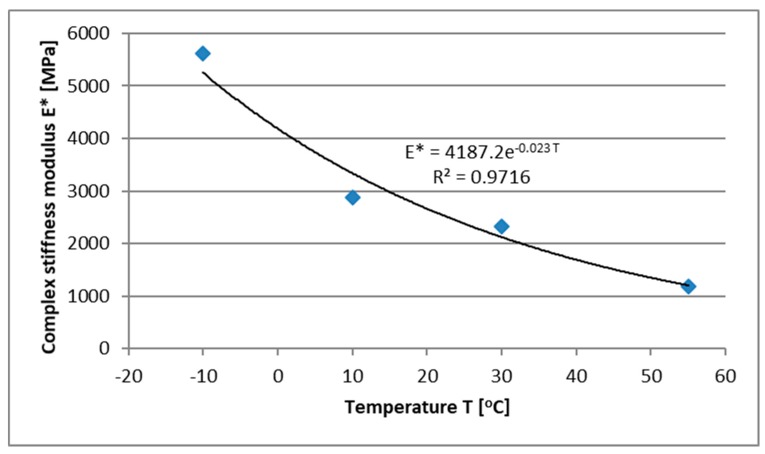
Changes in the FAC mixture stiffness as a function of temperature.

On the basis of the analyses, the values of the complex stiffness modulus of the innovative material in a wide temperature range were determine, see Figure 4. The complex stiffness modulus was determined as a mean value form four samples for each applied temperature.

As the temperature increases, the stiffness of FAC mixtures decreases. For a set average annual temperature of +10 °C, the complex stiffness modulus of the FAC mixture is about 2883 MPa. Temperature changes affect the stiffness of FAC mixtures, but the gradient of changes is smaller compared to conventional asphalt mixtures.

In the fatigue life analysis, the 4-point 4PB bending method was used—the dynamic method at constant deformation. To demonstrate the nature of the work of the FAC recycled material and to determine the fatigue criteria, tests were carried out for FAC mixtures in various environmental conditions. 

**Figure 5:**
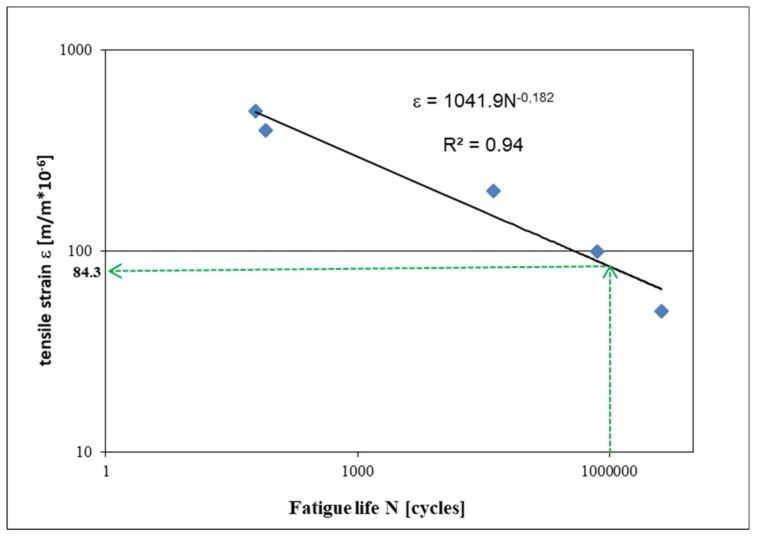
FAC mixture fatigue curve—permanent deformation method.

It was assumed that the number of load cycles N_f/50_, to achieve a decrease in the complex stiffness modulus to 50% of the initial value, is equivalent to the destruction of the sample, then the test was also discontinued. The N_f/50_ criterion applies to mineral-asphalt mixtures according to [38]. After the fatigue tests, data was obtained that allowed the estimation of the FAC mix fatigue curve—Figure 5. The fatigue curve is a relationship of fatigue life (on a logarithmic scale) as a function of the applied load value (strain). Fatigue life was determined as a mean value form six samples for each applied load strain. 

**Figure 6:**
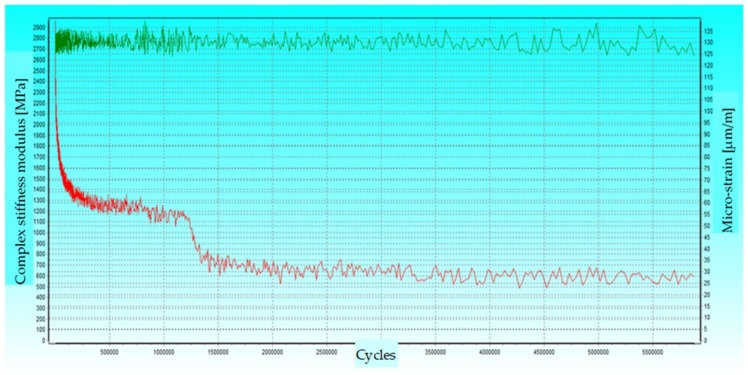
The course of the FAC mixture fatigue test at standard load.

From the slope of the fatigue curve, it should be concluded that the destructive deformation for 1 million load cycles is equal ε_6_ = 84.3 × 10^−6^ m/m. The different nature of the decrease in the value of the complex stiffness modulus was found in comparison with typical asphalt mixtures during fatigue tests, see Figure 6. The level of the applied load is marked in green, while the red color indicates the decrease in the value of the complex stiffness modulus.

FAC mixtures lose a significant part of the complex stiffness modulus relatively quickly and may be characterized by local decreases in stiffness, but they are still able to carry the given load in the form of strain. This is because microcracks appear in the analyzed material, which do not disqualify it for use in the layers of the road pavement base, in which such cracks are acceptable [39,40]. All tested mixtures had a similar character in the fatigue test.

Due to the presented conditions of FAC mixtures, the allowable decrease in the complex stiffness modulus should be modified to a level of approx. 30%, compared to the initial value. For the modified fatigue criterion N_f/30_, the number of load cycles, to obtain a decrease in the complex stiffness modulus to the level of 30% of the initial value, the level of destructive strain was estimated in the millionth cycle of loading ε_6_.

**Figure 7:**
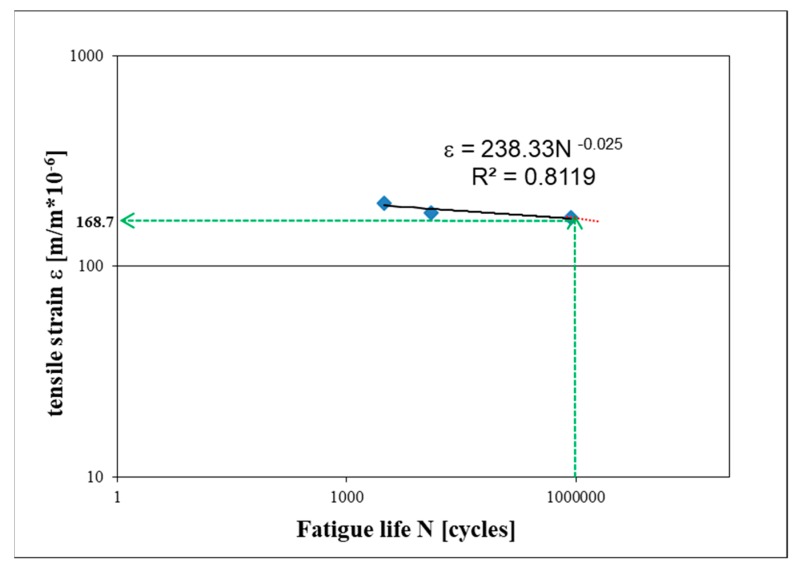
Fatigue curve—FAC.

Additional fatigue life tests of FAC mixtures were carried out for this reason, with the modified fatigue criterion N_f/30_. To obtain fatigue curves, fatigue tests were performed using strain levels: ε = 200 × 10^−6^ m/m, ε = 180 × 10^−6^ m/m, ε = 170 × 10^−6^ m/m. On the basis of the fatigue characteristics of the material, destructive strains were estimated in a millionth load cycle ε_6_. The results of laboratory tests are shown in Figure 7.

On the basis of the obtained fatigue characteristics (fatigue equation - Figure 7), the permissible level of destructive strain ε_6_ in a millionth cycle of loading was estimated at level ε_6_ = 168.7 × 10^−6^ m/m. Similar analyses were made for all FAC mixtures characterized by a different cement content and different asphalt content. The obtained values of destructive strain ε_6_ are presented in Table 5.

While analyzing the fatigue lives of tested mixtures it was set that with increasing the amount of asphalt and decreasing the amount of cement the mixtures became more flexible—the destructive strain ε_6_ increased. 

Using the conducted fatigue tests, the final form of the fatigue equation described by Equation (1), taking into account changes in foamed asphalt and cement content was obtained.

(1) $$\varepsilon =\varepsilon_{6}\cdot {\left(\frac{E_{10}}{E_{T}}\right)}^{0.78721}\cdot {\left(\frac{N_{\frac{f}{30}}}{10^{6}}\right)}^{\left(-0.57403\cdot A+0.64234\cdot C\right)},$$

where:

ε—acceptable tensile strain,

ε_6_—tensile strain at which the sample is destroyed after 10^6^ load cycles in the following test conditions: bending of a 4-point beam, temperature +10 °C, frequency 10 Hz,

N_f/30_—number of load cycles to achieve a decrease in the complex stiffness modulus to 30% of the initial value [-],

A—“A” asphalt content [%], 

C—“C” asphalt content [%],

E_10_—stiffness modulus of the mixture at +10 °C,

E_T_—stiffness modulus of the mixture at temperature T.

Laboratory tests and the developed fatigue equation can be used to predict the fatigue life of structural layers of road pavement, taking into account the shifting factors.

## 4. Test Section

### 4.1. Road Pavement Technology

**Figure 8:**
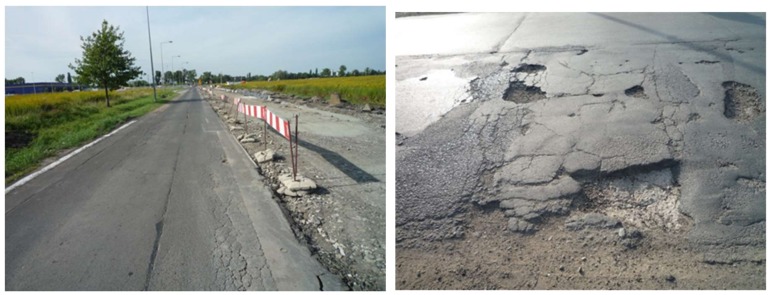
A section of the existing road—west lane.

Because the embedded materials in the existing road pavement have lost their bearing capacity and fatigue durability, it was proposed to make this pavement in cold recycling technology with existing materials and to make a FAC mixture on their basis. The C3A3 mixture was built into the base layer of the road pavement. Before finishing the test section, the road had numerous damages, which are shown in Figure 8. 

**Figure 9:**
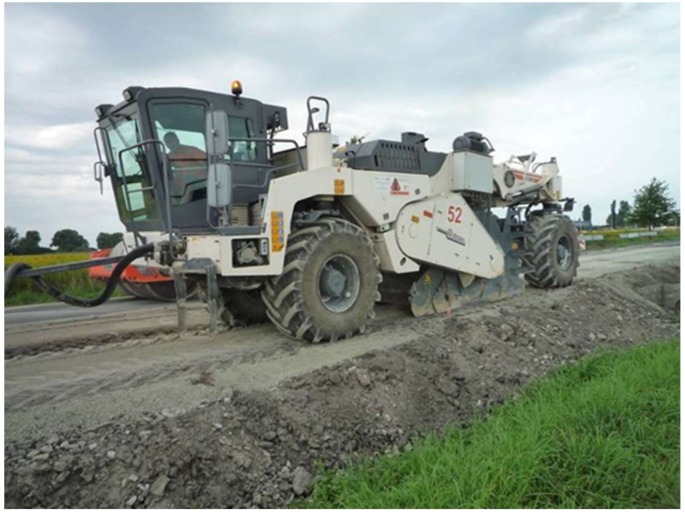
Recycling on-site—crushing and mixing of ingredients.

A mobile deep recycler was used to produce the recycled FAC mixture—Figure 9.

To verify the compaction of the base layer, the compaction index was checked. This indicator was determined by comparing the bulk density of samples formed from the FAC mixture in the laboratory with the bulk density of samples out of the finished pavement layer. The compaction index was 0.98, which is a satisfactory value for the base layers [25]. 

**Figure 10:**
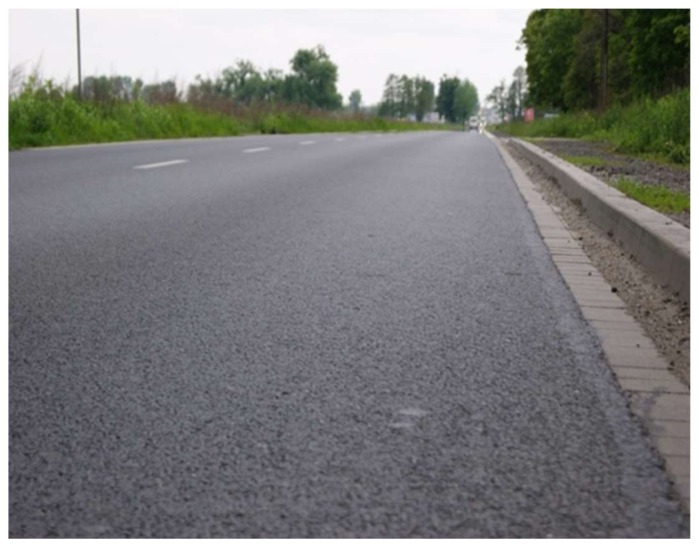
Wearing course layer, SMA type.

After constructing the recycled layers, the surface of the experimental section was finished with asphalt layers: a base layer and a base course layer of asphalt concrete (with a high stiffness modulus (ACWMS) and a wearing course layer of the SMA mixture, see Figure 10.

The design of the innovative road pavement structure assumed the following layering:

- SMA wearing course layer, 4 cm thick,
- ACWMS 16 base course layer, 8 cm thick,
- base made of ACWMS 16, 12 cm thick,
- base made of FAC mix, 20 cm thick,
- ground soil.

According to [38], all implemented mineral-asphalt mixtures met the design requirements for the layers of flexible pavement road constructions in Poland. As a result of the applied technology of the road base made of FAC-type mixture, the road durability forecasting was carried out before the road traffic admission. For this purpose, measurements of deflections of the pavement were carried out, along with the identification of layer modules and the subgrade.

### 4.2. Identification of Layer Modules

The measurements of pavement deflections were done on the street pavement using an FWD (Falling Weight Deflectometer). It is a device that induces a force impulse using a falling weight onto a measuring plate (through a specially designed spring system). The set of displacements determined on a given measuring stand creates the so-called “displacement bowl”, which is then used to identify modules of layers and the subrade. Measurements of deflections made by FWD were carried out during the construction of the section on the layers: the FAC layer, the ACWMS layer, and the SMA wearing course layer.

**Figure 11:**
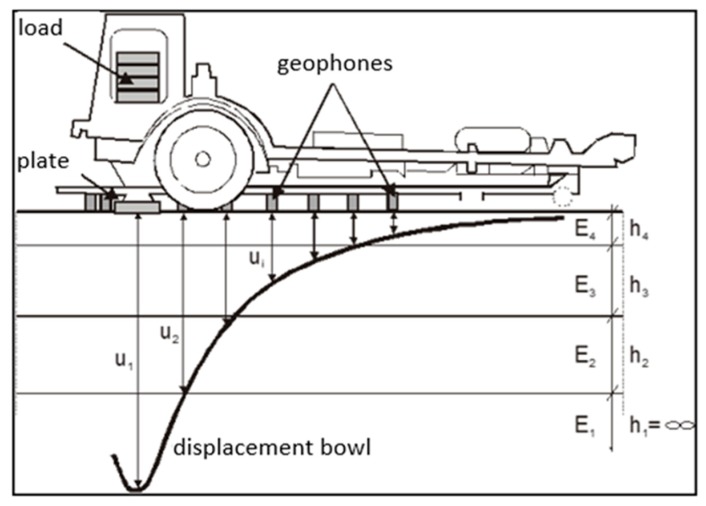
Measurement diagram done with an FWD deflectometer.

**Figure 12:**
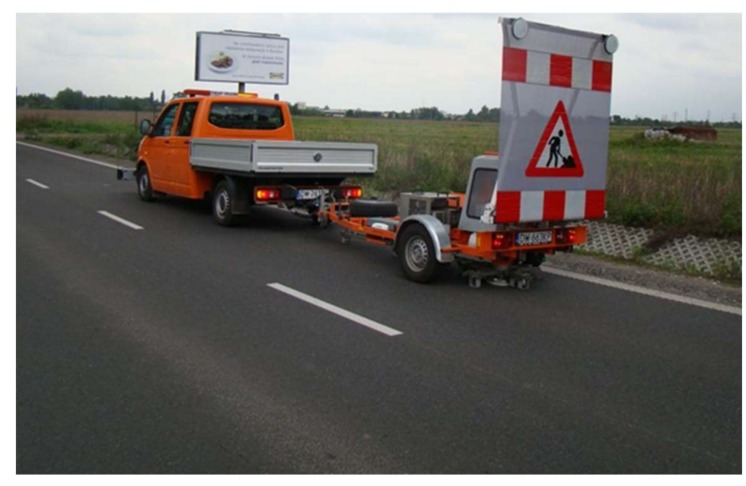
View of the FWD device during the test.

During pavement tests, displacement was measured at the following distances from the load axis: d1 = 0.0; d2 = 0.2; d3 = 0.3; d4 = 0.45; d5 = 0.6; d6 = 0.9; d7 = 1.2; d8 = 1.5; d9 = 1.8 m. The tests were carried out at different temperatures. Figure 11 shows a diagram of deflection testing using an FWD deflectometer. Figure 12 shows a view of the FWD deflectometer during the testing of this pavement.

**Figure 13:**
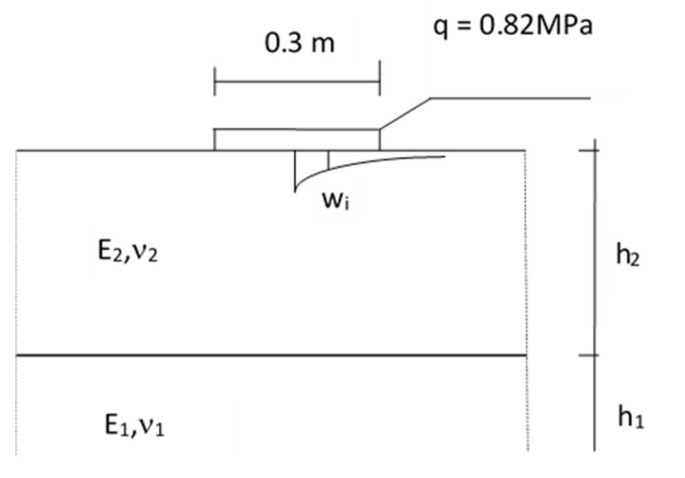
Calculation model of the tested pavement construction.

The results of deflection measurements were used to estimate the layer modules and the subgrade modules of the road pavement construction. The calculation model presented in Figure 13 was adopted for the identification calculations of the modules of the FAC layer. It is an elastic two-layer system, i.e., a layer arranged on the elastic half-space.

Particular layers model the layout of the pavement structure. The h_2_ layer models FAC, the h_1_ layer—an improved subgrade. The FAC layer is described by the E_2_ stiffness modulus and Poisson’s ratio ν_2_. The subgrade is described by the modulus of elasticity E_1_ and Poisson’s ratio ν_1_. The thicknesses were assumed by the in-depth identification h_2_ = 0.20 m. It was assumed that Poissonߣs ratio did not have a significant impact on the state of stress and strains and was assumed to be constant, i.e., ν_2_ = 0.3 and the factor ν_1_ = 0.35.

The essence of identification is to minimize the objective function described by Equation (2):

(2) $$\Delta =\frac{\sqrt{\frac{F}{k}}}{\frac{\sum_{j=1}^{k}w_{j}}{k}}$$

where:

(3) $$F=\sum_{j=1}^{k}{\left(w_{j}-u_{j}\right)}^{2}$$

*w_j_*—theoretical deflections calculated in the model,
*u_j_*—measured deflections,
*k*—the number of deflections measured at one point, forming the deflection bowl.

Of course, the number of layers n should be smaller than the number of k points forming the deflection bowl. Calculations were made on the basis of the CZUG program [41]. 

As a result of identification, the following values of modules (Ei) of the FAC layer and subgrade were obtained. Measurements of deflections on the FAC layer were made for different temperatures: −2 °C, +10 °C, +25 °C, +32 °C.

The obtained modulus values for the subgrade and the FAC layer are summarized in Table 6 for a 95% level of confidence.

After the FAC layer was laid and FWD tests were done, mma layers were laid, after which the deflection bowl measurements were again carried out using an FWD deflectometer.

**Figure 14:**
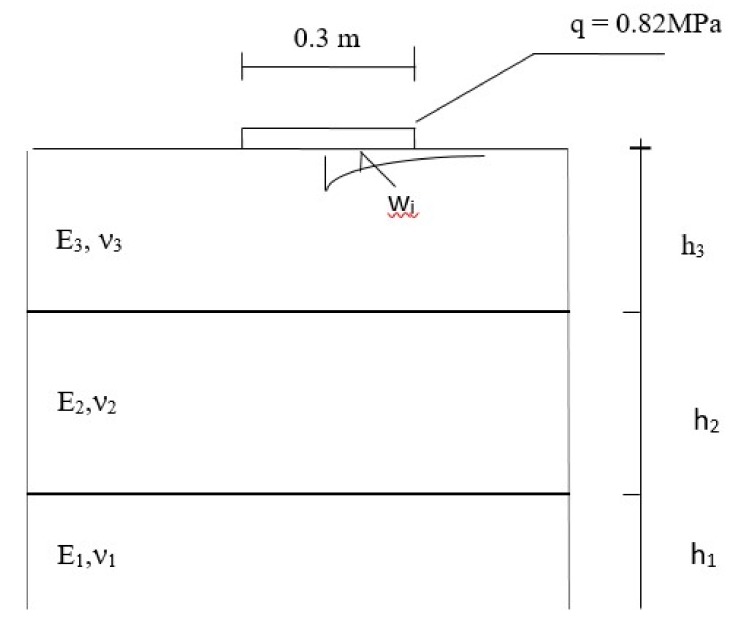
Calculation model of the tested pavement structure.

Figure 14 shows a model of the pavement structure after laying layers of mma. It is a three-layer system. Two layers are arranged on a half-space. The h_3_ layer is a layer with mma described by the E_3_ stiffness modulus and Poisson’s ratio ν_3_. The h_2_ layer is the FAC layer described by the E_2_ stiffness modulus and Poisson’s ratio ν_2_. The subgrade is described by the E_1_ modulus and Poisson’s ratio ν_1_. It was assumed that h_3_ = 0.24 m, h_2_ = 0.20 m, ν_3_ = ν_2_ = 0.35, and ν_1_ = 0.3.

The module values were calculated on the basis of the deflection bowl measurements using the FWD deflectometer and optimization calculations. The results are summarized in Table 7. Measurements were taken at the approx. temperature +10 °C.

**Figure 15:**
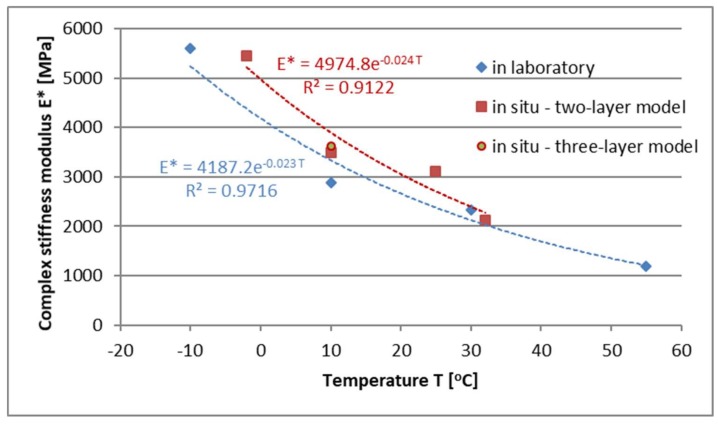
Values of modules in the laboratory and in situ.

Figure 15 summarizes the test results and compares the FAC mixture modules obtained in the laboratory and in situ layers.

When analyzing the results from Table 6 and Table 7 and Figure 15 the stiffness modulus value determined in the laboratory is comparable with the modules of the material used in situ. As a result of the analyzes, a good correlation between field and laboratory tests was obtained in the analyzed range of temperatures. The conversion factor of the modules "k" determines the relationship (4), which describes the ratio of the stiffness modules defined in the laboratory to the values of the field modules, as a function of temperature.

(4) $$k=0.8417\cdot e^{0.001\cdot T},$$

where:

k—module conversion factor [-],

T—FAC layer temperature [°C]. 

The “k” conversion factor takes values from 0.84–0.87 depending on the temperature—Table 8.

### 4.3. Fatigue Durability of Pavement Layers Structure

Using the obtained module values and the model presented in Figure 14, the fatigue life of the structure was calculated for the designed thicknesses. Equation (5), developed by the authors, describes the criterion for the FAC mixture:

(5) $$\varepsilon =\varepsilon_{6}\cdot {\left(k\right)}^{0.78721}\cdot {\left(\frac{N_{\frac{f}{30}}}{10^{6}}\right)}^{\left(-0.57403\cdot A+0.64234\cdot C\right)}\cdot f_{1}\cdot f_{2}\cdot f_{3}$$

where: 

ε—strain in the FAC layer,

ε_6_—strain at millionth load cycle, 0.000168 was adopted,

N_f/30_—number of load cycles to achieve a decrease in the complex stiffness modulus to 30% of the initial value,

A—percentage of new asphalt in the FAC layer, 4.16% was adopted,

C—percentage of cement in the FAC layer, 3.38% was adopted,

k—a conversion factor of the modules defined in the laboratory to the modules in situ, for a temperature of 10 °C,

*f*_1_—a shift factor dependent on stiffness of FAC mixture, range between 0.8–1.0, was adopted 0.81,

*f*_2_—a shift factor dependent on bearing capacity of subgrade, range between 0.8–1.0, was adopted 0.83,

*f*_3_—a shift factor dependent on heterogeneity of FAC mixture, range between 0.8–0.95, was adopted 0.92.

For the identified modules and model from Figure 14, strains at the bottom of the FAC layer were calculated. ε = 0.0000412 was obtained. Using Equation (5), N = 29,500,000 axles of 115 kN were calculated (fatigue life). The required minimal number of load axles for the road pavement is equal 12,000,000 axles 115 kN.

## 5. Conclusions

The conducted analyses for FAC mixtures (foamed asphalt mixtures with cement) showed that:

- the proposed conglomerates can be used for incorporation into road base layers.
- Identification tests of embedded layers confirmed the results of laboratory tests. The presented results indicate that the innovative technology used allows for the use of recycled materials, which significantly speeds up repairs.
- During the research work, a technology for modernizing degraded road pavements was developed. The cold recycling technology based on a FAC mixtures was implemented to provide adequate load-bearing capacity and fatigue durability of new road pavement construction.
- The results of in-situ tests in the field of module evaluation correlated with the results of laboratory tests.
- A new fatigue criterion for FAC mixtures and a correlation factor for stiffness modules determined in the laboratory and in situ were developed.
- Stiffness and bearing capacity tests showed that the pavement construction made with innovative technology, i.e., recycled material bonded with foamed asphalt and cement, has sufficient bearing capacity and fatigue life.
- As a result of the bearing capacity analyses, it was found that the layers of the test section meet the requirements for safe exploitation and their durability is satisfactory. Thus, the pavement on the test section could be put into exploitation.
- Laboratory tests of the stiffness modulus and fatigue durability developed fatigue criteria, and correlation factors can be used to estimate the durability of future road pavement structures based on the base layers of FAC mixtures.

## Figures and Tables

**Table 1:** Grading of the mineral mixture.

Sieve Size	Reclaimed Asphalt (Test Section)	Stone Base (Test Section)	Crushed Granite Stone (Test Section)	Material for Improving Gradation (Aggregate Mining)	Passes	Bottom Curve	Upper Curve
**[mm]**	**[%]**	**[%]**	**[%]**	**[%]**	**[%]**	**[%]**	**[%]**
63.0	0.00	0.00	0.00	0.00	100.00	100	100
3.5	0.00	12.05	0.00	0.00	87.95	70	100
25.0	0.00	4.25	1.42	0.00	82.29	65	100
20.0	0.00	4.24	1.82	0.00	76.23	60	100
16.0	0.17	1.57	1.41	0.09	72.99	55	100
12.8	0.11	0.99	1.28	0.94	69.68	49	93
8.0	0.29	1.28	2.21	6.15	59.75	40	84
6.3	0.65	0.37	0.96	3.13	54.64	35	78
4.0	2.60	0.45	1.58	4.90	45.10	25	68
2.0	2.39	0.33	2.78	4.40	35.19	15	50
0.85	2.96	0.41	5.39	4.05	22.38	10	37
0.42	1.74	0.35	3.16	1.56	15.58	8	28
0.30	0.78	0.22	1.59	0.54	12.46	4	18
0.15	0.79	0.37	2.69	0.75	7.86	3	11
0.075	0.44	0.29	1.42	0.79	4.93	3	8
<0.075	2.17	1.15	0.60	1.00	0.00	0	0
Total	15.10	28.30	28.30	28.30	100.00		

**Table 2:** Composition of FAC mixtures.

No.	Material Name	Share in MM	Share in FAC			
		[%]	[%]			
			Recipe No.			
			C3A3	C4A3	C3A5	C2A3
1	Reclaimed asphalt pavement (RAP)	15.10	13.14	12.98	12.83	13.29
2	Stone base	28.30	24.62	24.34	24.05	24.90
3	Crushed granite stone	28.30	24.62	24.34	24.05	24.90
4	Material for improving gradation	28.30	24.62	24.34	24.05	24.90
5	CEM I 42.5	C	3.38	4.38	3.38	2.38
6	Foamed asphalt	A	3.50	3.50	5.50	3.50
7	Water	W	6.13			

**Table 3:** Laboratory research program.

No.	Examination	Temperature	Curing Period in the Air
		[°C]	[days]
1	Density of FAC mix according to [33]	20	7, 14, 28
2	Bulk density of the FAC mixture according to [34]	20	7, 14, 28
3	Air void content according to [35]	20	7, 14, 28
4	Compressive strength according to [26]	25	7, 14, 28
5	Marshall stability and flow according to [32]	60	7, 14, 28

**Table 4:** Properties of the FAC mixture (C3A3).

Analyzed Feature	Samples Compacted in the Laboratory		
	Curing Conditions		
	7 Days	14 Days	28 Days
Density [g/cm^3^]	2.520	2.522	2.525
Bulk density [g/cm^3^]	2.218	2.220	2.226
Air void content [%]	11.75	11.69	11.66
Marshall stability [kN]	10.06	11.33	11.84
Marshall flow [mm]	0.84	0.78	0.76
Compressive strength [MPa]	1.62	2.21	2.45

**Table 5:** Fatigue durability of FAC mixtures.

Material Name	Share in MM	Share in FAC			
	[%]	Recipe No.			
		C3A3	C4A3	C3A5	C2A3
CEM I 42.5	C	3.38	4.38	3.38	2.38
Foamed asphalt	A	3.50	3.50	5.50	3.50
Destructive strain _6_ m/m*10^−6^		168.7	171.1	187.1	198.6

**Table 6:** List of identified module values.

Temperature during the Test [°C]	E1—Subgrade Modulus [MPa]	E2—FAC Layer Modulus [MPa]
−2	132	5450
+10	135	3490
+25	140	3120
+32	128	2120

**Table 7:** List of identified modules for the temperature +10 °C.

E1—Subgrade Modulus [MPa]	E2—FAC Modulus [MPa]	E3—Mma Modulus [MPa]
138	3620	16,250

**Table 8:** List of conversion factor "k" values.

Temperature T [°C]	Factor k [-]
−2	0.84
10	0.85
25	0.86
32	0.87
